# Removal of fluoride ions from aqueous solution by metaettringite

**DOI:** 10.1371/journal.pone.0265451

**Published:** 2022-03-14

**Authors:** Atsushi Iizuka, Hsing-Jung Ho, Akihiro Yamasaki

**Affiliations:** 1 Center for Mineral Processing and Metallurgy, Institute of Multidisciplinary Research for Advanced Materials, Tohoku University, Sendai, Miyagi, Japan; 2 Department of Materials and Life Science, Faculty of Science and Technology, Seikei University, Musashino, Tokyo, Japan; Lappeenranta-Lahti University of Technology (LUT University), FINLAND

## Abstract

Fluoride contamination is a major problem in wastewater treatment. Metaettringite (which has previously shown enhanced anion adsorption) was investigated as a possible adsorbent to remove fluoride from low-concentration solution (25 mg-F/L). The fluoride removal properties of ettringite and metaettringite were first compared at pH 10, and metaettringite was found to be more effective. The dominant reaction mechanism for fluoride adsorption in metaettringite was found to be recrystallization of metaettringite by rehydration; this was accompanied by precipitation of calcium fluoride. The adsorption kinetics followed the pseudo-second order model. Metaettringite was also able to remove fluoride effectively in low pH environment (i.e., at pH 3.5). The influence of coexistence of sulfate ions in solution on the fluoride removal performance was investigated, and a small decrease in performance was noted. The residual fluoride concentrations obtained with higher doses of metaettringite were lower than those specified by the Japanese effluent standard (non-coastal areas: 8 mg-F/L; coastal areas: 15 mg-F/L). The fluoride removal capacity of metaettringite was compared with those of other solid materials. The observed maximum capacity was 174.7 mg-F/g-metaettringite. In the case of high fluoride concentration solution, the main removal mechanism will be changed to calcium fluoride precipitation. In general, metaettringite is regarded as promising material for fluoride removal in wastewater treatment.

## 1. Introduction

In the past, compared with the attention given to heavy metals and organic pollutants, the importance of fluoride contamination in effluent has usually been underestimated. However, people have recently begun to notice that the impact of water and soil pollution from fluoride is a serious problem. Although humans require trace amounts of fluoride [1], exposure to excess fluoride has negative impacts on humans and livestock [2]. Unfortunately, many industries produce wastewater that exceeds allowable fluoride levels for human consumption or environmental discharge [3]. Thus fluoride removal is a critical part of water treatment.

High fluoride content is commonly found in wastewaters from industries including the mining [4], semiconductor [5, 6], fertilizer [7, 8] and photovoltaic [9] sectors. This means that a broad range of options to remove fluoride and prevent fluoride contamination are needed. The conventional treatment is to add chemicals (usually Ca(OH)_2_) to precipitate fluoride as CaF_2_. However, CaF_2_ precipitation is difficult to treat in low-fluoride-concentration water since the final fluoride concentration can only be reached by 10–20 mg/L due to the solubility of CaF_2_ [10]. Recent reviews have summarized and detailed the development of various fluoride removal technologies, including membrane [11], adsorption [12], electrocoagulation [13] and coagulation-precipitation [14] techniques. Among them, the use of adsorbents seems one of the most promising methods for fluoride removal [10, 15], because it is cost-effective and easy to implement; moreover, it can have high removal capacity and can be reused [10]. Functional metal oxides with nanostructures, such as porous nano-MgO [16–18] and La@MgAl nanocomposites [19], show superior fluoride adsorption performance. Recently, various adsorbents, such as shells [20–22], food waste biochar [23], sepiolite [24], dolomite [25], and crushed concrete [26], have been developed for fluoride removal. In the adsorption field, the research trend is toward studying the potential of by-products and waste as adsorbents for fluoride removal.

Ettringite (Ca_6_Al_2_(SO_4_)_3_(OH)_12_·25–26H_2_O) is a mineral that forms during the hydration of Portland cement, and is typically found in cementitious material; however, it is also attracting significant attention as an adsorbent because of its anion-exchange ability [27]. The SO_4_^2-^ ions in ettringite can be ion-exchanged with other anions, such as B(OH)_4_^-^ [28–31], F^-^ [32, 33], PO_4_^3-^ [34], AsO_4_^3-^ [35, 36] and CrO_4_^2-^ [37]. Besides, adsorbents containing ettringite can be prepared from wastes, such as waste concrete [35, 38], concrete sludge [32, 39] and by-product gypsum [40]. Based on the above points, ettringite seems promising for F^-^ adsorption.

In our preliminary study [29], we modified ettringite by heat treatment at relatively low temperature to form metaettringite and found that the boron-adsorption performance was greatly enhanced. This finding suggests potential for adsorption of other anions using metaettringite, but thus far studies of removal of other anions by metaettringite are still limited. Therefore, the adsorption performance of metaettringite should be investigated further. Because metaettringite can be prepared from waste, it is in line with current research trends, and thus it is important to determine the fluoride removal performance of this adsorbent; therefore, this study was conducted as a basic investigation of this topic.

## 2. Materials and methods

### 2.1. Materials

We have previously investigated the preparation of metaettringite from ettringite in detail [29] and prepared the metaettringite used in this study in a similar way. Ettringite was first synthesized from Ca(OH)_2_ (99%, Wako Pure Chemical Industries, Osaka, Japan), Al_2_(SO_4_)_3_ (85%, Wako Pure Chemical Industries, Osaka, Japan) and CaSO_4_·2H_2_O (98%, Wako Pure Chemical Industries, Osaka, Japan). 0.6 mol Ca(OH)_2_ and 0.1 mol Al_2_(SO_4_)_3_ were each dissolved in 1 L distilled water. Then, the Ca(OH)_2_ solution (slurry-like solution) and Al_2_(SO_4_)_3_ solution were mixed with saturated CaSO_4_·2H_2_O solution (1 L) for 4 h at 80°C. Then, the slurry was filtered by pressure filtration and the solids were freeze-dried overnight before being crushed with a muddler. The ettringite structure was confirmed by X-ray diffraction (XRD; RINT2000, Rigaku, Tokyo, Japan). Metaettringite was then prepared by calcination of ettringite at 65°C for 72 h, as described in Eq 1. The particle size of metaettringite was selected in the range of 53 to 106 μm in this study.


$$\begin{array}{l} 3\text{CaO}\cdot {\text{Al}}_{2}{\text{O}}_{3}\cdot 3{\text{CaSO}}_{4}\cdot m{\text{H}}_{2}\text{O}\Rightarrow \\ \;3\text{CaO}\cdot {\text{Al}}_{2}{\text{O}}_{3}\cdot 3{\text{CaSO}}_{4}\cdot n{\text{H}}_{2}\text{O}+\left(m–n\right)\;{\text{H}}_{2}\text{O},\;(31≦m≦32) \end{array}$$ (1)

### 2.2. Fluoride-ion removal experiments

Table 1 shows the standard conditions used for fluoride-ion removal experiments. The aqueous solution was prepared from guaranteed reagent-grade sodium fluoride (NaF, 99%, FUJIFILM Wako Pure Chemical Corporation, Osaka, Japan) and distilled water. The initial fluoride concentration was set at 25 mg/L to simulate the fluoride concentration in mine wastewater in Japan. Saturated calcium hydroxide solution and 1% nitric acid solution, prepared from calcium hydroxide (Ca(OH)_2_, 99.9%, FUJIFILM Wako Pure Chemical Corporation, Osaka, Japan) and nitric acid (HNO_3_, 70%, FUJIFILM Wako Pure Chemical Corporation, Japan), respectively, were used to adjust the pH to the desired value (as measured with a pH meter, AUT-701, TOA DKK, Tokyo, Japan). Selected amounts of prepared ettringite or metaettringite were added into 500 mL of fluoride solution in a beaker, stirred at 400 rpm, and allowed to react for 120 mins. Samples (15 mL) were taken using a syringe (SS-20LZ, Terumo, Tokyo, Japan) with a 0.45 μm syringe filter (25AS045AN, ADVANTEC, Tokyo, Japan) at reaction times of 0, 5, 10, 20, 30, 60, and 120 min. An Ion Chromatography System (ICS-1500, Dionex, Sunnyvale, CA, USA) was used to analyze the concentration of fluoride in solution. The eluent was a solution comprising 4.5 mmol/L Na_2_CO_3_ and 1.4 mmol/L NaHCO_3_, and the flow rate was 1.2 mL/min. Inductively coupled plasma atomic emission spectrometry measurements (ICP-AES, SPECTRO ARCOS EOP, SPECTRO, Germany) were used to analyze the concentrations of other elements.

**Table 1:** Standard conditions for adsorption experiments.

Conditions	
Initial fluoride concentration (mg/L)	25
Solution volume (mL)	500
Solution temperature (°C)	23±0.5
Adsorption time (min)	120
Stirring rate (rpm)	400
Particle size of sample (μm)	53–106

The performance of ettringite was compared with that of metaettringite at pH 10 and solid–liquid ratios of 1 and 10 g/L. The results of metaettringite were selected to investigate the adsorption kinetics using three conventional kinetics models (i.e., pseudo-first order model, pseudo-second order model, and intra particle diffusion model). The influence of the solid–liquid ratio of metaettringite was evaluated at pH 10; solid–liquid ratios of 1, 3, 5 and 10 g/L were used. The adsorption performance of metaettringite was also tested at pH 3.5, again using solid–liquid ratios of 1, 3, 5 and 10 g/L. To evaluate the influence of the presence of sulfate ions, 100 or 200 mg/L of sulfate were added in the form of sodium sulfate (Na_2_SO_4_, 99%, FUJIFILM Wako Pure Chemical Corporation, Osaka, Japan). Sorption isotherms were measured at pH 10 and a solid–liquid ratio of 5 g/L; the initial fluoride concentration was varied from 10 to 1000 mg/L.

## 3. Results

### 3.1. Performance comparison between ettringite and metaettringite

**Fig 1:**
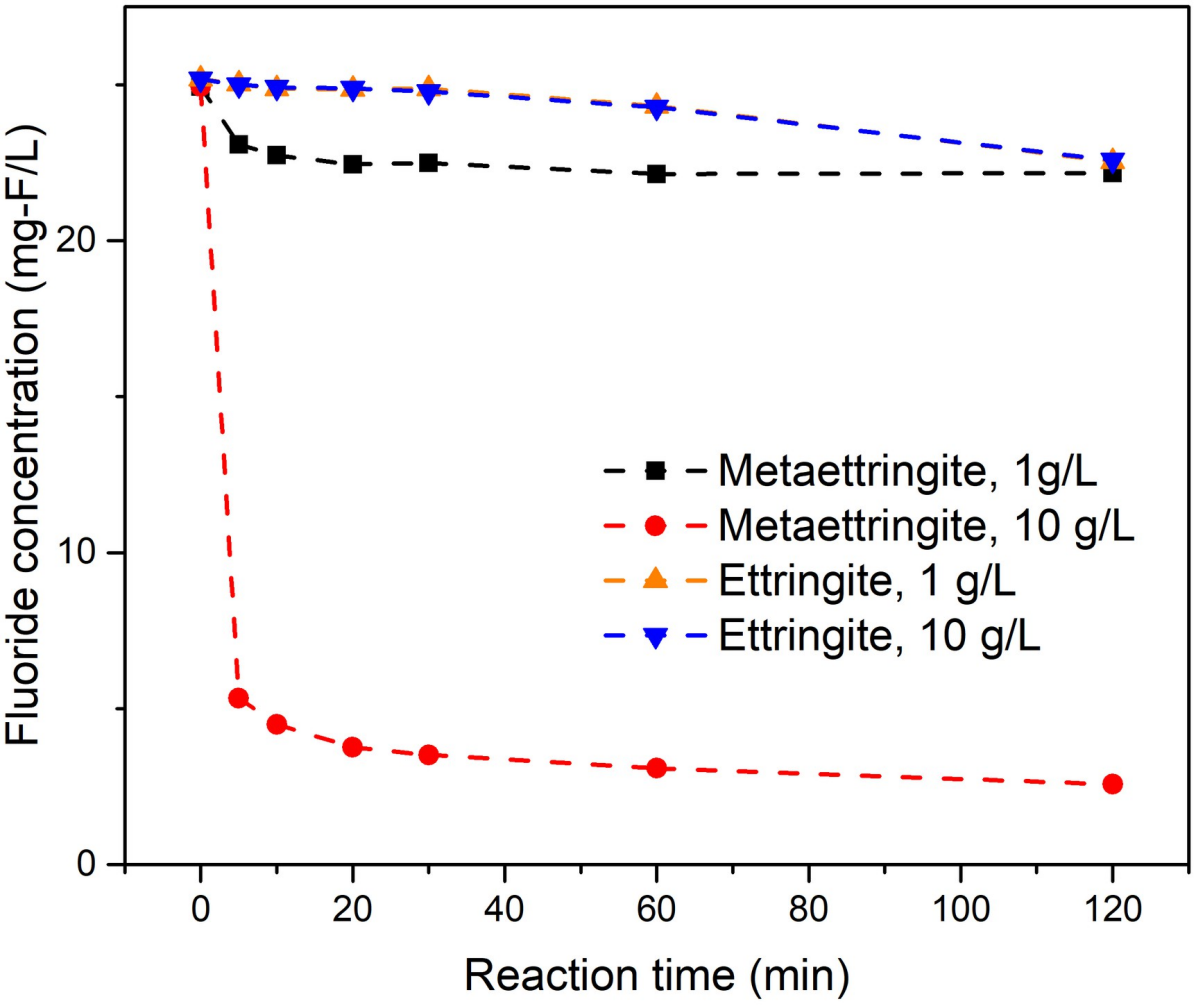
Comparison of time-dependent fluoride removal performance of ettringite and metaettringite.

Fig 1 compares the fluoride concentrations as a function of time for solutions treated with ettringite and metaettringite. The fluoride concentration curves obtained using ettringite are very similar at solid–liquid ratios of 1 and 10 g/L. At both solid–liquid ratios, the fluoride concentration obtained with metaettringite is lower than that obtained with ettringite. In the metaettringite tests, the fluoride concentration decreased immediately after the reaction started. In contrast, in the ettringite tests, the fluoride concentration remained quite constant over the first 30 mins. The final fluoride removal percentage of metaettringite was ~89.7% for a solid–liquid ratio of 10 g/L. As well as this, the ratio of the amount of solid phase after fluoride removal to the amount of solid initially added was higher for metaettringite (100.5%) than for ettringite (83.9%) at a solid–liquid ratio of 10 g/L. To determine the adsorption kinetics using metaettringite as the adsorbent, the results of metaettringite were fitted to the pseudo-first order model, pseudo-second order model, and intra particle diffusion model. The examined results are shown as Table 2. The correlation coefficient *R*^2^ values indicated that among the three models, the pseudo-second order model provided the best agreement (*R*^2^ = 0.998 and ~1.000). The overall pseudo-second order rate constant (*k*_2_, g/mg/min) and adsorbate concentration at equilibrium (*q*_e_, mg/g) were obtained to evaluate the adsorption kinetics.

**Table 2:** Parameters of the kinetics models for fluoride adsorption using metaettringite at solid–liquid ratios of 1 and 10 g/L.

Dosage	1 g/L	10 g/L
Pseudo-first order model		
*q*_e_ (mg/g)	0.961	0.411
*k*_1_ (1/min)	0.044	0.041
*R* ^2^	0.767	0.687
Pseudo-second order model		
*q*_e_ (mg/g)	2.83	2.24
*k*_2_ (g/mg/min)	0.159	0.499
*R* ^2^	0.998	1.000
Intra particle diffusion model		
*k*_i_ (mg/g/min^1/2^)	0.212	0.148
*R* ^2^	0.552	0.340

### 3.2. Influence of solid–liquid ratio

**Fig 2:**
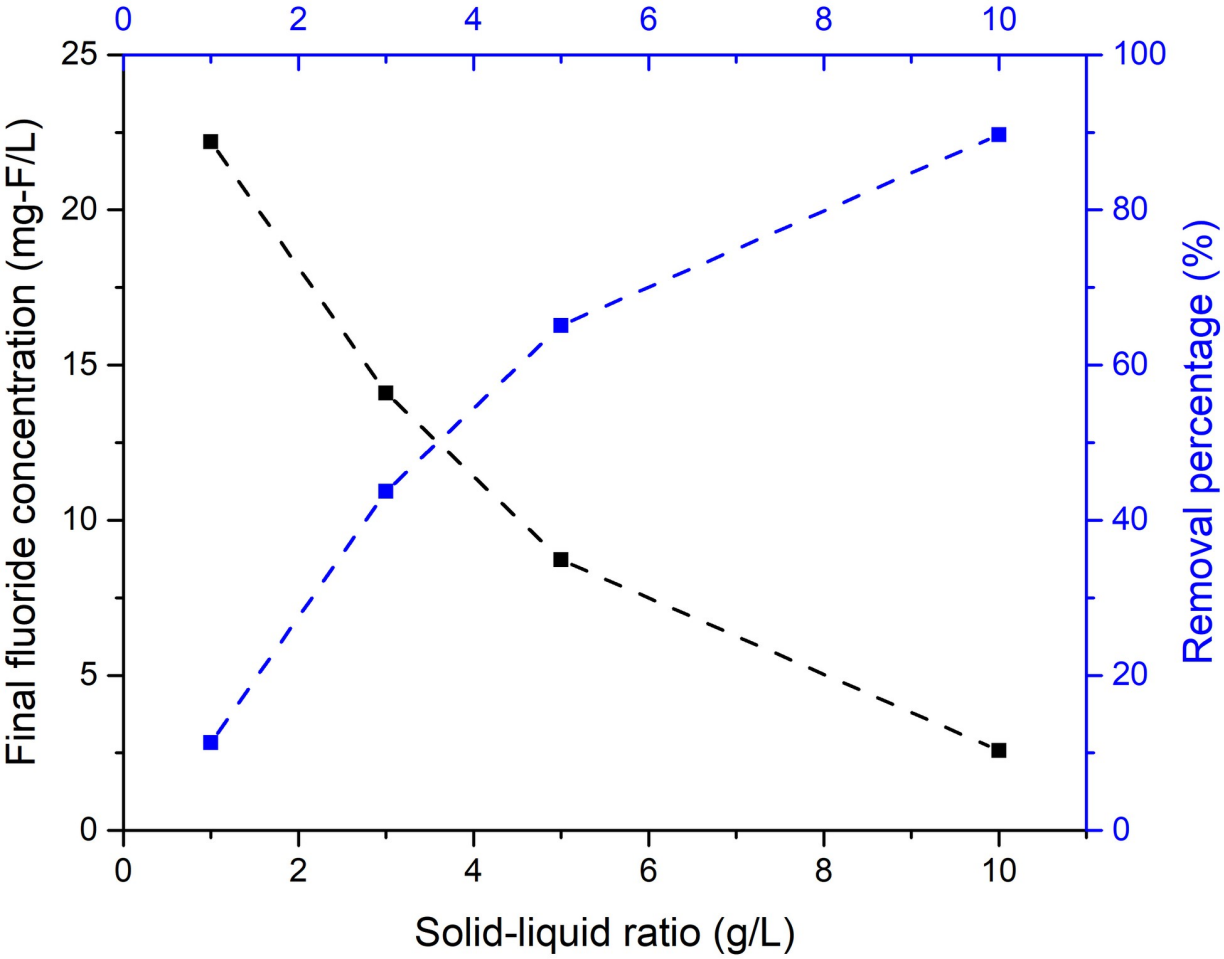
Final fluoride concentration and percentage of fluoride removed at various solid–liquid ratios for metaettringite.

Solid–liquid ratio is an important parameter that significantly affects the adsorption reaction. Fig 2 indicates that the fluoride removal percentage is greater at higher solid–liquid ratios. The fluoride removal percentages were 11.3%, 43.7%, 65.1% and 89.7%, and residual fluoride concentrations were 22.2, 14.1, 8.73 and 2.57 mg/L when solid–liquid ratios were 1, 3, 5 and 10 g/L, respectively.

**Fig 3:**
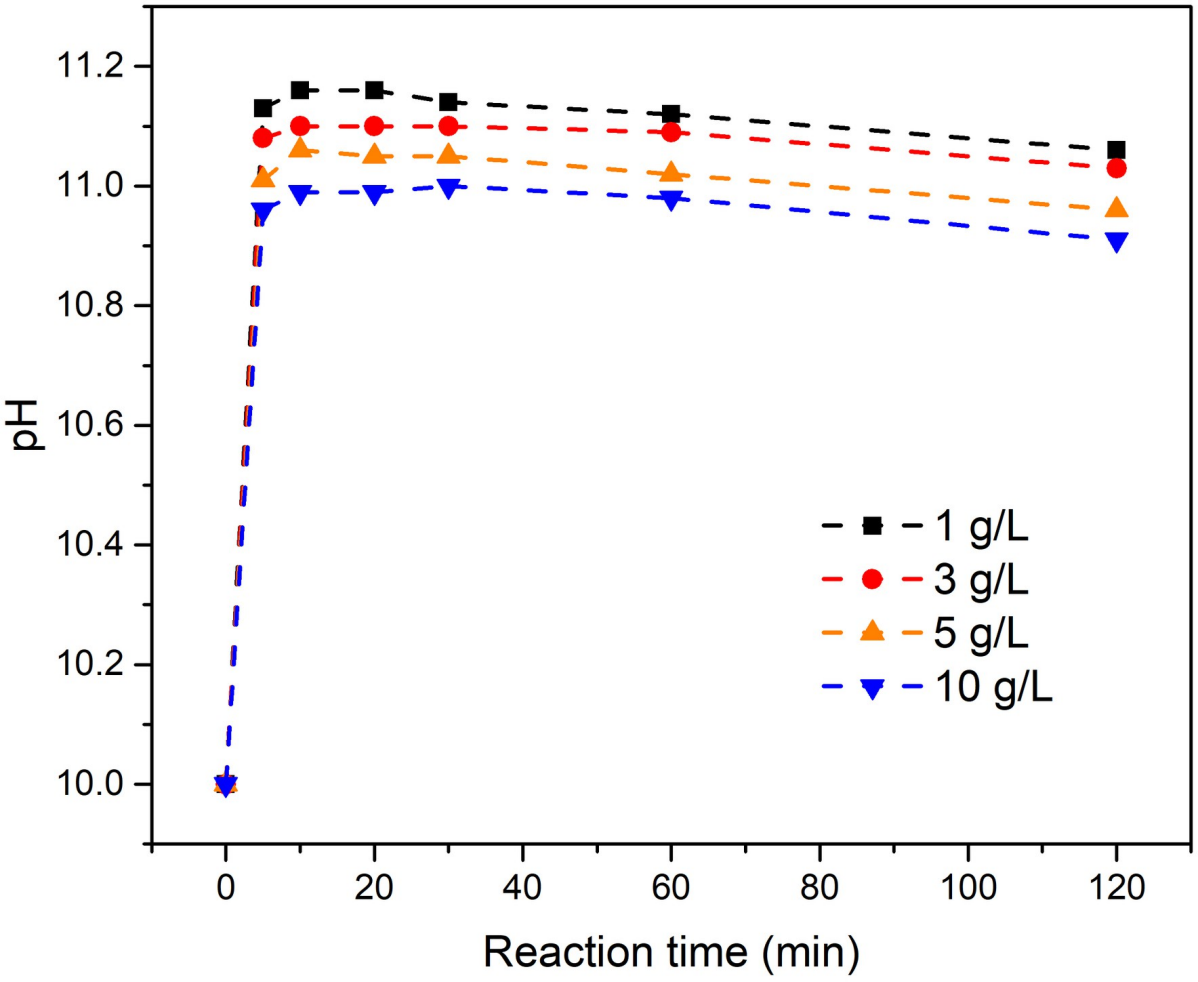
Variation of pH with time at various solid–liquid ratios for metaettringite.

Fig 3 shows the variation of pH with time at various solid–liquid ratios. The pH increased immediately and then leveled off. The pH trends were similar for various solid–liquid ratios and the final pH was 11±0.1 for all metaettringite concentrations studied.

**Fig 4:**
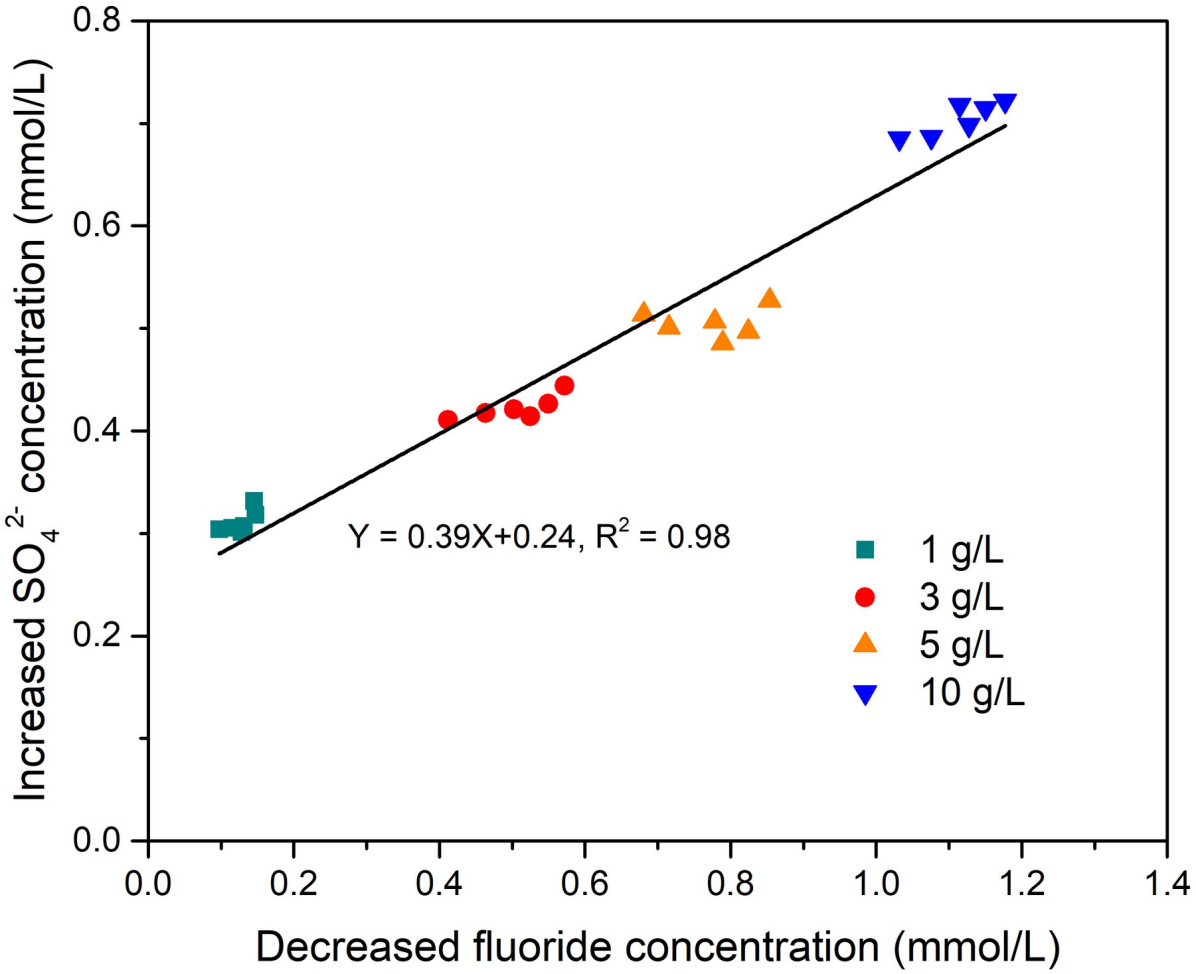
Relationship between decreased fluoride concentration and increased SO_4_^2-^ concentration in solution at different proportions of metaettringite.

To help understand the mechanism of fluoride removal by metaettringite, the relationship between decreased fluoride concentration and the increased SO_4_^2-^ concentration released into solution was determined for different solid–liquid ratios. The decreased fluoride concentration and increased SO_4_^2-^ concentration were defined as the concentration change of the initial and final concentration of the fluoride and SO_4_^2-^. Fig 4 shows that the slope of increased SO_4_^2-^ concentration to decreased fluoride concentration with increasing solid–liquid ratio is approximately 0.39 with an R^2^ of 0.98. The intercept of the linear fit is approximately 0.24.

### 3.3. Adsorption performance in low pH environment

**Fig 5:**
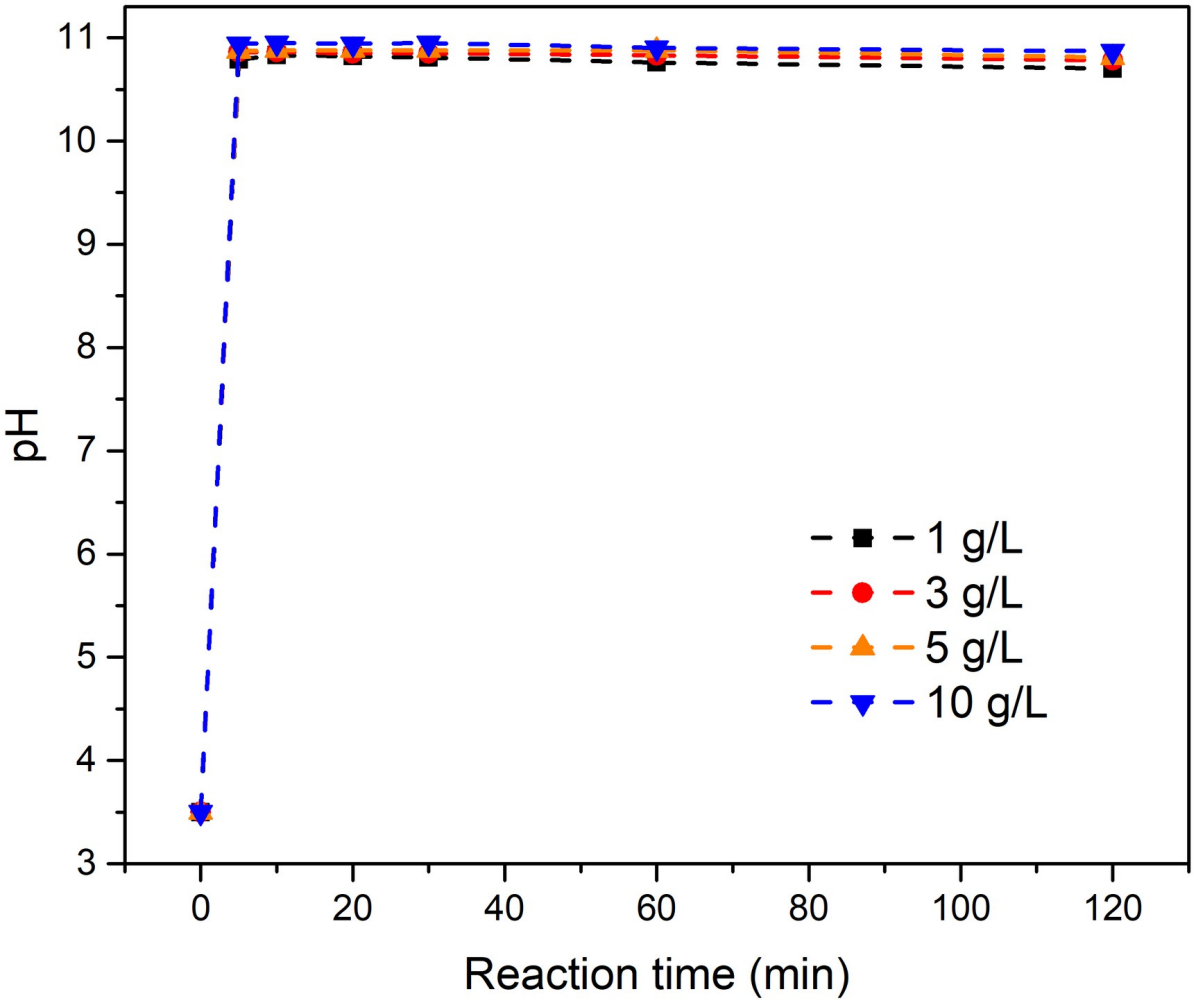
Variation of pH with time; initial pH of 3.5.

**Fig 6:**
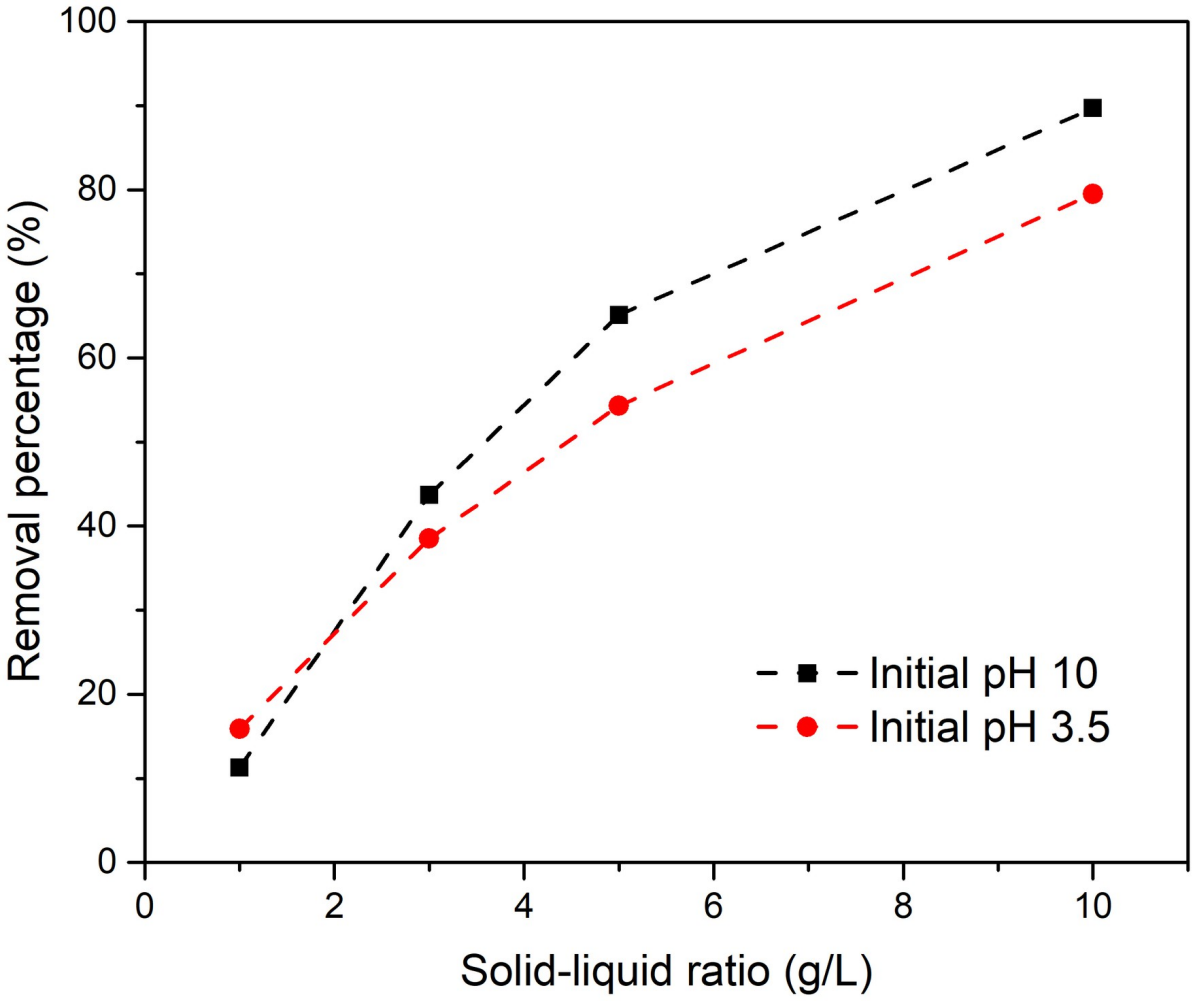
Effect of initial pH and solid–liquid ratio on percentage of fluoride removed; initial pH of 3.5 and 10.

Ettringite is unstable and partly dissolves in a low-pH environment [41]. Since metaettringite is obtained from ettringite, the adsorption performance of metaettringite in a low-pH environment must be determined. Removal tests were performed at an initial pH of 3.5 with solid–liquid ratios of 1, 3, 5 and 10 g/L. Fig 5 shows the variation of pH with time during this test; the pH immediately increased in all cases and reached 10.7–10.9 at 120 min. Fig 6 compares the fluoride removal percentages obtained for initial pH values of 10 and 3.5. The fluoride removal percentage is lower at an initial pH of 3.5 than at an initial pH of 10 for a solid–liquid ratio of 10 g/L. The final fluoride removal percentage for an initial pH of 3.5 and solid–liquid ratio of 10 g/L was 79.5%, corresponding to a final fluoride concentration of 5.12 mg/L.

### 3.4. Influence of coexistence of sulfate ions

**Fig 7:**
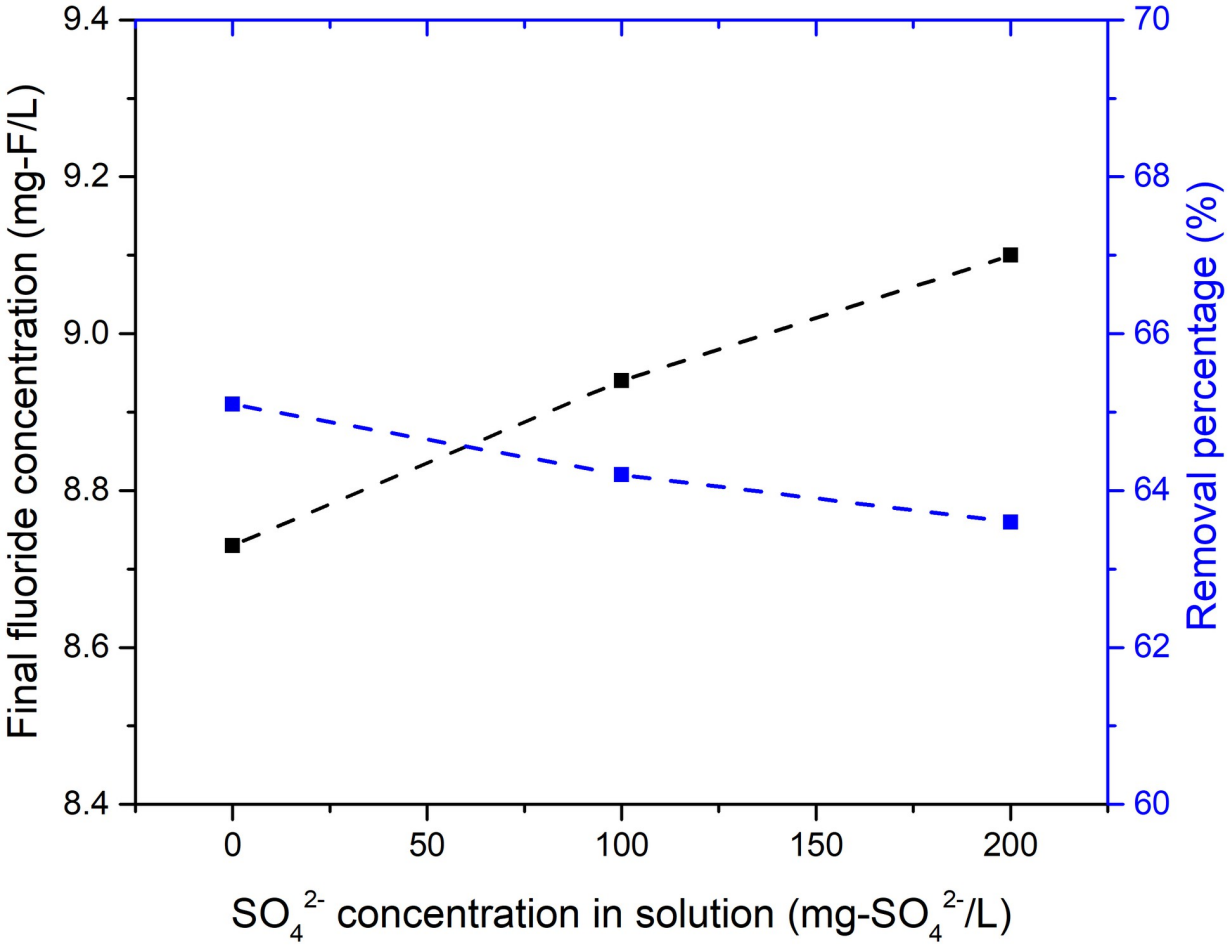
Influence of SO_4_^2-^ concentration in solution on fluoride adsorption.

In the compositions of mine wastewater, sulfate ions usually coexist with fluoride ions. Therefore, the influence of coexistence of sulfate ions was tested, as shown in Fig 7. The sulfate concentration was set at 0, 100 or 200 mg/L at pH 10 and a solid–liquid ratio of 5 g/L. A higher sulfate concentration in solution resulted in a slightly higher final fluoride concentration and lower fluoride removal percentage.

### 3.5. Adsorption isotherm

**Fig 8:**
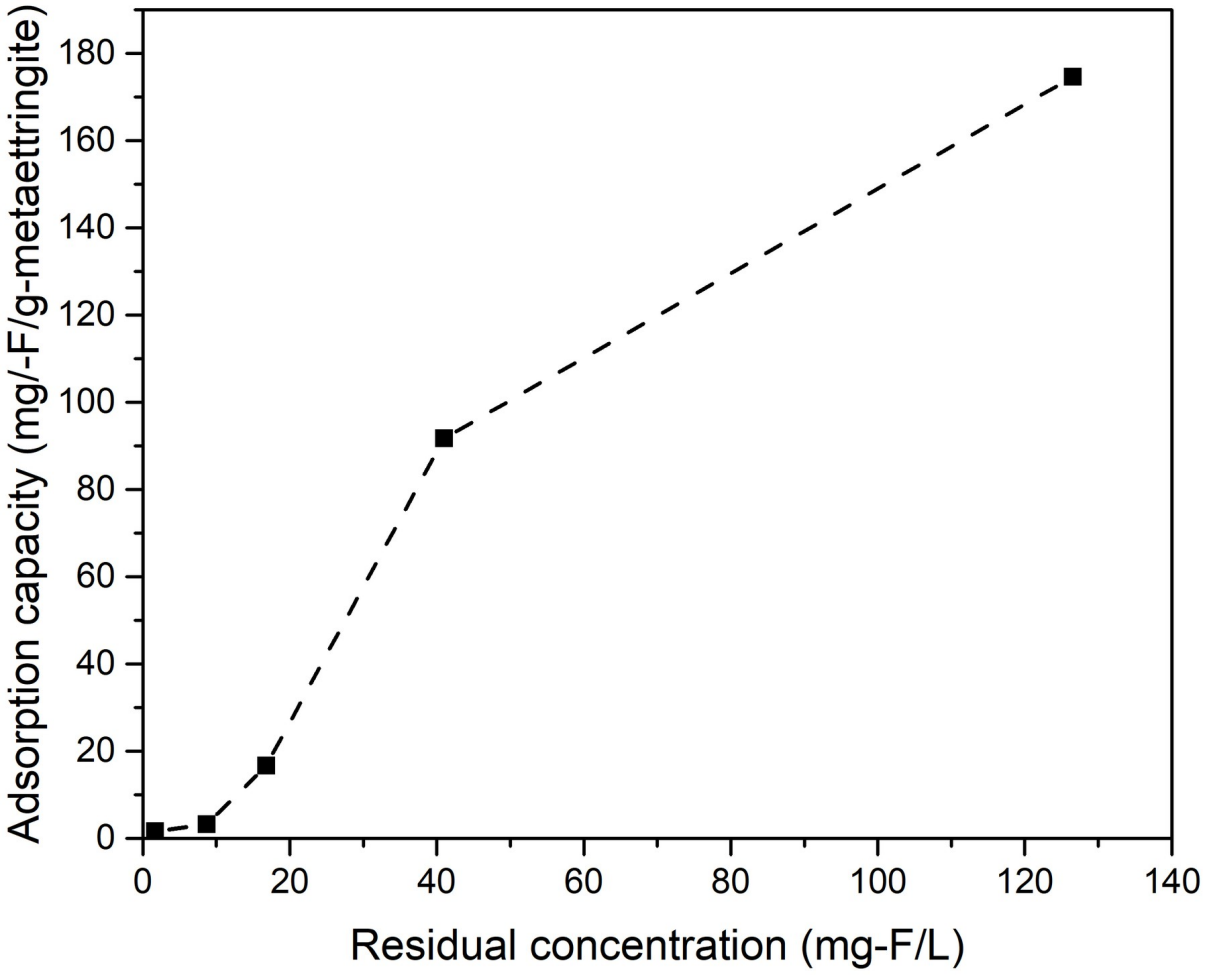
Adsorption isotherm of fluoride on metaettringite.

**Fig 9:**
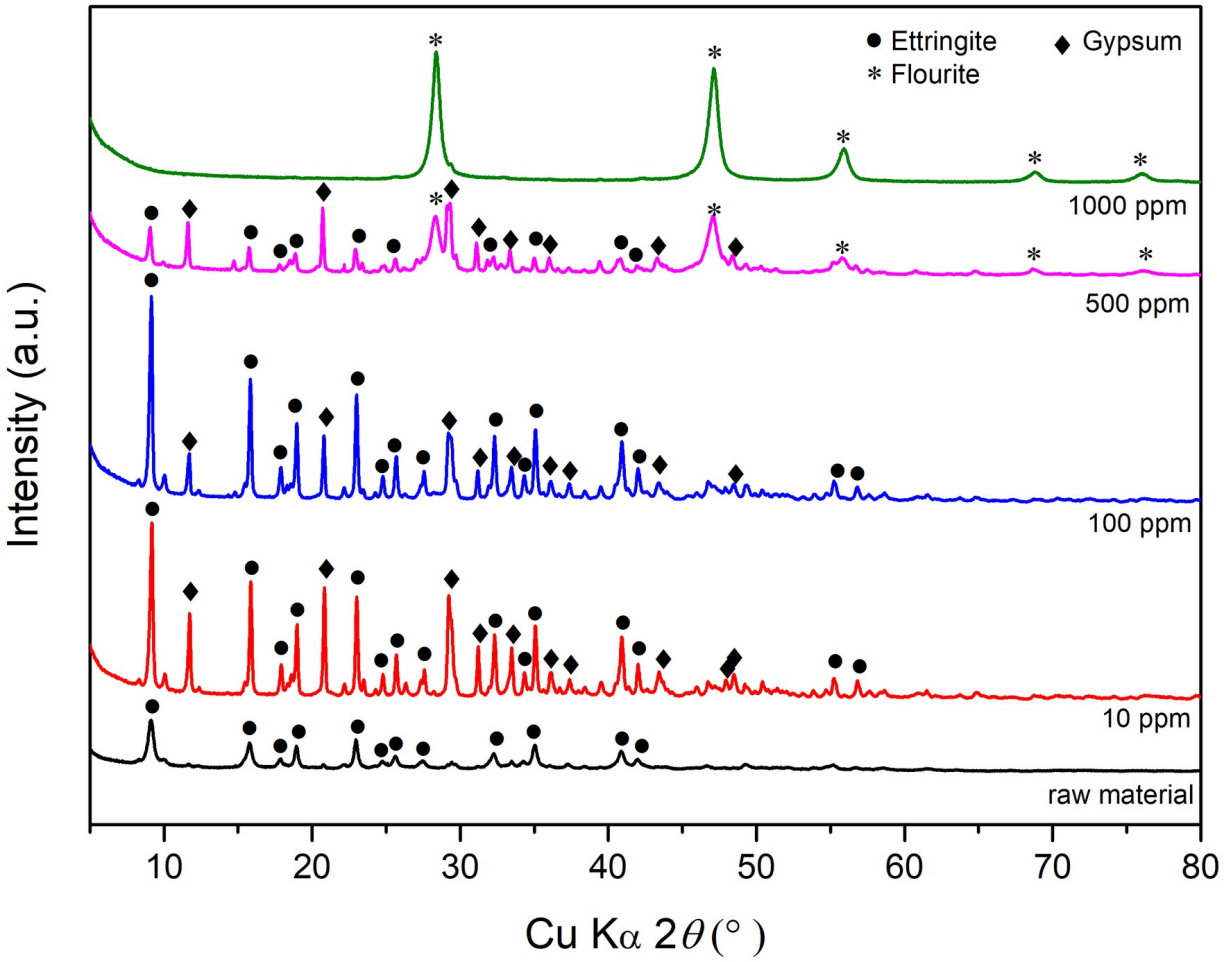
XRD patterns of metaettringite before and after fluoride removal with different initial fluoride concentration; symbol ● (ettringite, PDF No. 00-037-1776), * (fluorite, PDF No. 01-070-2782), ◆ (gypsum, PDF 00-006-0047).

To determine the fluoride removal performance of metaettringite, the apparent adsorption isotherm was investigated by varying the initial fluoride concentration at a fixed initial pH of 10 and solid–liquid ratio of 5 g/L. Fig 8 shows that the fluoride-adsorption uptake by metaettringite increases with increasing initial fluoride concentration, accompanied by increased residual fluoride concentration. At low initial fluoride concentration, fluoride ions are almost completely removed from the aqueous solution. The observed maximum adsorption capacity is 174.7 mg-F/g-metaettringite with a residual concentration of 126.6 mg-F/L of aqueous solution. Fig 9 shows the XRD patterns of metaettringite before and after fluoride removal with different initial fluoride concentration. It indicates that the peak of ettringite becomes stronger after fluoride removal and the peak of gypsum also appears. Besides, with the higher initial fluoride concentration, the peaks of ettringite and gypsum become weaker, and the peak of fluorite starts to appear while the initial fluoride concentration is greater than 500 mg-F/L. Moreover, conventional isotherm models (i.e., Langmuir isotherm and Freundlich models) were used to describe the isotherm behavior, as shown in Table 3. The results indicated that the two models did not adequately describe the observed adsorption, with correlation coefficient *R*^2^ values of 0.844 and 0.891 using the Langmuir isotherm and Freundlich models, respectively.

**Table 3:** Parameters of the isotherm models for fluoride adsorption using metaettringite.

Langmuir isotherm model	
*q*_max_ (mg/g)	25.0
*b* (L/mg)	0.041
R^2^	0.844
Freundlich isotherm model	
*K*_f_	0.604
*n*	0.841
*R* ^2^	0.891

## 4. Discussion

We investigated fluoride removal by metaettringite. The ettringite structure is composed of columns (i.e., {Ca_6_[Al(OH)_6_]_2_.24H_2_O}^6+^) and intercolumn spaces that contain substitutable sulfate ions and water molecules. The mechanism for adsorption of fluoride by ettringite is ion exchange of sulfate ions with fluoride ions. It has been suggested that the ion exchange of sulfate ions in ettringite might only occur in the surface layer [30]. Compared with ettringite, metaettringite produced a far lower final fluoride concentration in the solution. The ideal adsorption capacities per mole should be the same for ettringite and metaettringite, since their contents of sulfate ions are the same. In fact, the key reason for the greater fluoride-adsorption performance of metaettringite is that metaettringite has greater accessibility for ion exchange. The ion-exchange rate is enhanced by dehydration. According to our preliminary study [29], two different types of water molecules in ettringite are removed at 65°C (i.e., 2 water molecules from the intercolumn space and 12 water molecules weakly bound in additional vertices of the trigonal prisms). With treatment at elevated temperature, ettringite becomes amorphous metaettringite. During the fluoride removal experiment, the recrystallization of metaettringite by rehydration is the main reaction mechanism. The ion exchange in metaettringite is considered to be dual synergistic ion exchange, [31] which not only takes place in the surface layer during recrystallization of metaettringite through rehydration, but also in the intercolumn space. Additionally, a high specific surface area is achieved in metaettringite. Therefore, the adsorption performance of metaettringite is much greater than that of ettringite. In addition, the ratio of the amount of solid phase after fluoride removal to the initial feed amount is higher for metaettringite than for ettringite. This can be attributed to the rehydration of metaettringite to form an ettringite structure, as previously found in our preliminary study, where we used ettringite and metaettringite for boron removal [29]. Moreover, the adsorption kinetic was determined by three conventional models. Among the three models, pseudo-second order model shows good fits, evidenced by the highest correlation coefficient, close to 1, indicating adequately described the adsorption kinetics. At solid–liquid ratios of 1 and 10 g/L, the amounts of fluoride adsorbed at equilibrium (*q*_e_) were respectively 2.83 and 2.24 mg/g, which were similar to the observed q_e_ values of 2.77 and 2.23 mg/g. The good fit of the pseudo-second order model indicates that the adsorption reaction rate is controlled by chemisorption, which is the hypothesis of this model [42]. It also indicates that the adsorption capacity is proportional to the active sites on the adsorbent surface, which is consistent with the reaction mechanism of metaettringite recrystallization by rehydration.

At a higher solid–liquid ratio, greater fluoride removal percentage and lower remaining fluoride concentration were obtained. This is because more sulfate ions from metaettringite are accessible to react with fluoride ions through ion exchange. According to Fig 2, when the solid–liquid ratio increases to 3 mg/L, the final fluoride concentration meets the Japanese effluent standard for coastal areas (15 mg-F/L) [43]. When the solid–liquid ratio increases to 5 mg/L, the final fluoride concentration meets the Japanese effluent standard for non-coastal areas (8 mg-F/L) [43]. The final pH was ~11 at all solid–liquid ratios. It is worth mentioning that the pH range where ettringite is stable is 10.5–13 [41], which implies that the recrystallized ettringite after rehydration is stable at all solid–liquid ratios tested.

The relationship between decreased fluoride concentration and increased SO_4_^2-^ concentration in solution with different solid–liquid ratios was determined. Theoretically, two F^-^ can be ion-exchanged with one SO_4_^2-^ in the ettringite structure, as shown in Eq 2.


$$\begin{array}{l} {}[{\text{Ca}}_{6}[\text{Al}{\left(\text{OH}\right)}_{6}{]{}_{2}\cdot 24{\text{H}}_{2}\text{O}]}^{6+}{\left[3\left({\text{SO}}_{4}{}^{2-}\right)\right]}^{6-}+6{\text{F}}^{-}\Leftrightarrow \\ \;[{\text{Ca}}_{6}[\text{Al}{\left(\text{OH}\right)}_{6}{]{}_{2}\cdot 24{\text{H}}_{2}\text{O}]}^{6+}{\left[6\left({\text{F}}^{-}\right)\right]}^{6-}+3{\text{SO}}_{4}{}^{2-} \end{array}$$ (2)

However, according to Fig 4, the slope is ~0.39, which indicates that the increase of removed fluoride ions is higher than the theoretical increase of SO_4_^2-^ (Removed F^-^: SO_4_^2-^ = 2.56: 1). This indicates that the fluoride removal mechanism is not only ion exchange with SO_4_^2-^, but also includes precipitation of calcium fluoride. According to the concentrations of calcium (over 70 mg/L in all cases) and fluoride in solution, the value of ion product exceeds the solubility product constant of CaF_2_ (*K*sp = 3.9 × 10^−11^ mol^3^/L^3^ at 25°C [44]), so calcium fluoride precipitation is expected. The increase of solution pH and calcium concentration after the addition of metaettringite is due to the partial dissolution of metaettringite and recrystallized ettringite. The intercept of the linear fit in Fig 4 is about 0.24, which indicates that the released amount of SO_4_^2-^ is about 0.24 mmol/L (23.5 mg/L) even though no fluoride has been removed. This may be because of partial dissolution of metaettringite, resulting in a certain amount of SO_4_^2-^ from metaettringite being released into solution, which is consistent with earlier results in this study.

Metaettringite was determined to be able to remove fluoride ions at pH 3.5. The pH immediately increased from 3.5 to over 10.5 at all solid–liquid ratios tested. The removal percentages were lower than those obtained when the initial pH was 10. This may be attributed to the partial dissolution of metaettringite and recrystallized ettringite. The dissolution of metaettringite can be described by Eq 3. However, the final pH is 10.8±0.1, which is in the stability region of ettringite [41]. The fluoride removal performance of metaettringite is still able to meet the effluent standard for non-coastal areas (8 mg-F/L) in Japan [43] even though the initial pH was acidic, demonstrating that the metaettringite has the potential to remove fluoride ions in acidic solution.


$$3\text{CaO}\cdot {\text{Al}}_{2}{\text{O}}_{3}\cdot 3{\text{CaSO}}_{4}\cdot n{\text{H}}_{2}\text{O}\;(n≦32)\Rightarrow {\text{Al}}_{2}{\text{O}}_{3}+3{\text{CaSO}}_{4}+3{\text{Ca}}^{2+}+6{\text{OH}}^{-}+\left(n-3\right){\text{H}}_{2}\text{O}$$ (3)

Coexistence of sulfate ions in solution were found to slightly decrease the fluoride-removal percentage. This can be explained by the relative ion-exchange priorities of sulfate and fluoride ions, and is consistent with the results of a previous study [41]. When sulfate ions exist in an aqueous solution, they will be incorporated into ettringite in preference to other ions. Thus, for fluoride removal, the coexistence of sulfate ions in solution will affect the adsorption performance and the competition of fluoride ions and sulfate ions must be considered, depending on their relative concentration in solution.

The adsorption isotherm was investigated by changing the initial fluoride concentration (10–1000 mg-F/L). The result implied that the maximum fluoride uptake capacity was not reached during the test. Conventional adsorption isotherm models (i.e., Langmuir and Freundlich) were used to fit the results of this study. The correlation coefficient was better with the Freundlich isotherm model (*R*^2^ = 0.891) in the plot of log(*q*_e_) versus log(*C*_e_) than with the Langmuir isotherm model (*R*^2^ = 0.844) in the plot of 1/*q*_e_ versus 1/*C*_e_, indicating that, of the two models, the Freundlich isotherm model was more suitable for describing the experimental results, although *R*^2^ was not greater than good. This indicates that the adsorption of fluoride is likely controlled by the heterogenous nature of the adsorbent with an exponential distribution of adsorption energy on the surface sites instead of monolayer adsorption at homogenous sites [45]. The reason for the poor fits using the two conventional isotherm models is related to the actual adsorption reaction, which is metaettringite recrystallization by rehydration accompanied by precipitation of calcium fluoride; therefore, the actual reaction did not obey the model assumptions. Considering the ion product with solubility product constant of calcium fluoride, the adsorption capacity was contributed by ion exchange and precipitation of calcium fluoride. The XRD patterns of residual solids proves the occurrence of recrystallization of metaettringite and the precipitation of calcium fluoride and gypsum. The precipitation of gypsum shows that the fact of variation of SO_4_^2-^ is not only from ion exchange with F^-^ but also precipitation with Ca^2+^. Besides, under the condition of low initial concentration of fluoride, the fluoride is mainly removed by ion exchange during the recrystallization of metaettringite by rehydration. It is proved by the occurrence of strong peak of ettringite under cases of 10 and 100 mg-F/L. However, when the initial concentration of fluoride is high, the fluoride is mainly removed by the precipitation of calcium fluoride. This is because the abundant amount of fluoride promotes fluoride ions to react with calcium ions and precipitate as calcium fluoride instead of ion-exchanging with sulfate ions. In contrast, when the initial concentration of fluoride is low, because of the limitation of calcium fluoride solubility, the fluoride can only be effectively removed by ion exchange, resulting in no peak of calcium fluoride.

Compared with results of previous studies (Table 4), the fluoride-adsorption capacity found for metaettringite is high. In general, cementitious solid adsorbents use the ettringite component in cement to remove fluoride. However, in this study, the ettringite was changed to metaettringite, which enhanced the fluoride uptake capacity, resulting in greater adsorption performance. Moreover, most of the adsorbents prepared from by-products or waste require thermal treatment (315–800°C) to activate their performance. Compared with the temperature required for those adsorbents, that required for thermal treatment in this study is low (65°C), indicating that relatively low cost and low power consumption can be achieved for the thermal treatment in this work. Treatment at relatively high temperatures is required to activate those above-mentioned adsorbents, thus transforming calcium-related compounds into calcium oxide or biomass into metal-biochar composites. However, the use of metaettringite, usually prepared from waste concrete and concrete sludge, does not require the conversion of calcium-related compounds. In addition, adsorbents made from ettringite can be regenerated by heat treatment, improving the economic benefit from an industrial point of view [31]. Anion-loaded ettringite may also be directly reused as a filler in concrete production [34]. However, fluorine is unacceptable in cement and concrete production. It is difficult to reuse F-loaded metaettringite, which is the same as other adsorbents, although a relatively high adsorption capacity can be realized. In summary, metaettringite is a promising material to remove fluoride from aqueous solutions.

**Table 4:** Comparison of the fluoride removal capacities of metaettringite and other solid materials prepared from by-products or waste.

Material	Capacity (mg-F/g)	Remarks	Ref.
Metaettringite (this study)	1.67–174.7	Initial fluoride concentration: 10–1000 mg/L, initial pH = 10, reaction time = 120 min. The sorbent was prepared by thermal treatment at 65°C.	
Solid adsorbent derived from calcined *Patinopecten yessoensis* shells	159.62	Maximum adsorption capacity in the Langmuir model. The sorbent was prepared by thermal treatment at 800°C.	[20]
Solid adsorbent derived from eggshells	253.28	Maximum adsorption capacity in the Langmuir model. The sorbent was prepared by thermal treatment at 800°C. Initial fluoride concentration: 200–1000 mg/L.	[21]
Solid adsorbent derived from *Mytilus coruscus* shells	82.93	Maximum adsorption capacity in the Langmuir model. The sorbent was prepared by thermal treatment at 800°C. Initial fluoride concentration: 100–700 mg/L.	[22]
Solid adsorbent derived from aluminum-modified food waste biochar	123.4	Maximum adsorption capacity in the Langmuir model. The sorbent was prepared by thermal treatment at 315°C. Initial fluoride concentration: 10–900 mg/L.	[23]
Al-Cu oxide nanoparticles supported on steel slag waste	3.99	Maximum adsorption capacity in the Langmuir model. Initial fluoride concentration: 1–30 mg/L	[46]
Solid adsorbent derived from pine wood char	7.66	Maximum adsorption capacity in the Langmuir model. The sorbent was prepared by thermal treatment at 400°C. Initial fluoride concentration: 1–100 mg/L	[47]
Solid adsorbent derived from pine bark char	9.77	Maximum adsorption capacity in the Langmuir model. The sorbent was prepared by thermal treatment at 450°C. Initial fluoride concentration: 1–100 mg/L	[47]
Solid adsorbent derived from cement paste	79.6	Initial fluoride concentration: 400 mg/L	[48]
Solid adsorbent derived from cement paste	149	Initial fluoride concentration: 100 mg/L	[49]

## 5. Conclusions

The ability of metaettringite to adsorb fluoride ions from a low-concentration solution (25 mg-F/L) was investigated. The fluoride-removal performance of metaettringite was far greater than that of ettringite. The recrystallization of metaettringite by rehydration is the main reaction mechanism. The ion exchange by metaettringite is considered to be dual synergistic ion exchange, which not only occurs in the intercolumn space, but also in the surface layer during recrystallization of metaettringite through rehydration. The pseudo-second order model described the results obtained for fluoride removal by metaettringite, indicating that the rate-controlling step is likely chemisorption of the adsorbate on the adsorbent. Higher solid–liquid ratios, resulting in more accessible sulfate ions from metaettringite, produce higher fluoride-removal percentages. A further fluoride-removal mechanism (in addition to ion exchange) is the precipitation of calcium fluoride. The potential of metaettringite for fluoride removal in a low-pH environment was preliminarily investigated. Metaettringite can be applied even in acidic environments, although its performance will be diminished. The residual concentration in the low-pH solution was less than the effluent standard (8 mg-F/L) in Japan. The influence of coexistence of sulfate ions in solution was also investigated. Extra sulfate ions will slightly decrease the fluoride-removal percentage due to the priority of ion exchange. Besides, compared with the mechanism under low fluoride concentration, the main fluoride-removal mechanism of metaettringite with the high fluoride concentration solution was calcium fluoride precipitation promoted by the abundant amount of fluoride ions.

The fluoride-removal capacity of metaettringite was comparable with or better than those reported for other solid materials, showing that metaettringite has great capacity owing to its enhanced ion-exchange property. Metaettringite is an extremely promising material to treat fluoride-containing wastewater. The use of metaettringite for various types of wastewaters containing fluoride will be interesting.
